# Cigarette smoke alters inflammatory genes and the extracellular matrix — investigations on viable sections of peripheral human lungs

**DOI:** 10.1007/s00441-021-03553-1

**Published:** 2021-11-25

**Authors:** Helena Obernolte, Monika Niehof, Peter Braubach, Hans-Gerd Fieguth, Danny Jonigk, Olaf Pfennig, Thomas Tschernig, Gregor Warnecke, Armin Braun, Katherina Sewald

**Affiliations:** 1 Fraunhofer Institute for Toxicology and Experimental Medicine ITEM, Biomedical Research in Endstage and Obstructive Lung Disease Hannover (BREATH), Member of the German Center for Lung Research (DZL), Hannover, Germany; 2 Institute for Pathology, Hannover Medical School, Biomedical Research in Endstage and Obstructive Lung Disease Hannover (BREATH), Member of the German Center for Lung Research (DZL), Hannover, Germany; 3 KRH Klinikum Siloah-Oststadt-Heidehaus, Hannover, Germany; 4 Institute for Anatomy and Cell Biology, Saarland University, Homburg Saar, Germany; 5 Division of Cardiac, Thoracic, Transplantation, and Vascular Surgery, Hannover Medical School, Biomedical Research in Endstage and Obstructive Lung Disease Hannover (BREATH), Member of the German Center for Lung Research (DZL), Hannover, Germany; 6 Institute of Immunology, Hannover Medical School, Hannover, Germany

**Keywords:** COPD, Cigarette smoke condensate, Lipopolysaccharide, Precision-cut lung slices, Cytokines, Gene expression

## Abstract

Chronic obstructive pulmonary disease (COPD) is a complex chronic respiratory disorder often caused by cigarette smoke. Cigarette smoke contains hundreds of toxic substances. In our study, we wanted to identify initial mechanisms of cigarette smoke induced changes in the distal lung. Viable slices of human lungs were exposed 24 h to cigarette smoke condensate, and the dose–response profile was analyzed. Non-toxic condensate concentrations and lipopolysaccharide were used for further experiments. COPD-related protein and gene expression was measured. Cigarette smoke condensate did not induce pro-inflammatory cytokines and most inflammation-associated genes. In contrast, lipopolysaccharide significantly induced IL-1α, IL-1β, TNF-α and IL-8 (proteins) and IL1B, IL6, and TNF (genes). Interestingly, cigarette smoke condensate induced metabolism- and extracellular matrix–associated proteins and genes, which were not influenced by lipopolysaccharide. Also, a significant regulation of CYP1A1 and CYP1B1, as well as MMP9 and MMP9/TIMP1 ratio, was observed which resembles typical findings in COPD. In conclusion, our data show that cigarette smoke and lipopolysaccharide induce significant responses in human lung tissue ex vivo, giving first hints that COPD starts early in smoking history.

## Introduction

Chronic obstructive pulmonary disease (COPD) is a complex respiratory disorder characterized by poorly reversible progressive airway obstruction and abnormal airway inflammation (Hacievliyagil et al. 2006; Turato et al. 2001). It covers many pathological entities such as chronic bronchitis with permanent obstruction, respiratory failure leading to hypoxemia, and enlargement of airspaces to emphysema (Raherison and Girodet 2009). The most common symptoms of COPD are breathlessness, excessive sputum production, and chronic cough (WHO 2019). More than 300 million people worldwide have COPD. The disease is on the rise and will be the third leading cause of death in 2030 with 8.3 million death per year (WHO 2019). Common conception is that mainly cigarette smoke causes COPD. Cigarette smoke contains thousands of chemicals with a large number being antigenic, carcinogenic, cytotoxic, and mutagenic inducing pathological changes in the respiratory tract. The gaseous phase contains short life substances affecting the upper airways. The particulate or tar phase enters the lower respiratory tract and affects cells in the small airways and alveoli. More than 90% of COPD patients are smokers, but only 20% of the smokers develop COPD (Pauwels and Rabe 2004). The reasons why some smokers develop COPD, whereas other do not, are still unknown (GOLD 2004). Cigarette smoke induces pathological changes such as disrupted epithelial layers, ciliary dysfunction, and mucus hypersecretion of the respiratory tract in humans. It also affects a wide range of immunological functions of the respiratory tract and has been linked to increased susceptibility to infections (Danov et al. 2020; Gonçalves et al. 2011; Qiu et al. 2017; Sopori 2002; Sopori et al. 1994). Cigarette smoke–exposed macrophages express for example increased levels of lysosomal enzymes, oxygen radicals, myeloperoxidase, and elastase that damage the connective tissue (King et al. 2017; Reynolds 1987; Sopori 2002; Sopori et al. 1994). Cigarette smoke also impairs the ability of macrophages to phagocytose bacteria, to secrete pro-inflammatory cytokines (King et al. 1988; Martin 1977) and reduces the activity of natural killer cells (Ferson et al. 1979; Hogan et al. 2011; Qiu et al. 2017). Blood leukocytes of smokers are also suppressed in function, e.g., leading to reduced antibody formation and less adaptive responses to viral infections (Holt and Keast 1977; Sopori 2002; Tarbiah et al. 2019). The disruption of the epithelial layers recruits other immune cells such as macrophages and lymphocytes into lung tissue leading to airway inflammation. Airway inflammation is a characteristic for smokers and COPD patients, but its role during the onset of pathological changes is rather unknown. Most available information has been received from samples taken from the upper respiratory tract of smokers and COPD patients, e.g., by bronchial brushings (Barbers et al. 1987; Landi et al. 2011). Nevertheless, the major part of cigarette smoke, including the particulate phase, is mainly affecting the lower respiratory tract — a part of the lung that is not easily accessible. Our knowledge about first effects of cigarette smoke on the distal healthy lung is very limited. There are hints that alterations in the distal lung in smokers precede and likely lead to the emphysematous destruction of the alveolar structure in smokers and COPD patients (Shaykhiev and Crystal 2014). We are interested in understanding the role of inflammation and emphysema formation in the distal lung of healthy smokers. We hypothesize that acute exposure of human lung tissue to cigarette smoke condensate (CSC) induces pro-inflammatory responses and changes of the extracellular matrix (ECM) in human lung tissue of the lower respiratory tract overlapping with later changes in smokers and COPD patients. Therefore, we used healthy living tissue of peripheral distal lung of human donors and exposed it ex vivo to CSC.

In this study, we identified matrix-associated but not inflammation-associated genes induced in viable lung tissue from the distal lung by CSC. We show how CSC changes viability of human lung tissue, leads to altered pro-inflammatory cytokines, and changes in the extracellular matrix. In comparison, we analyzed changes induced by the well-known immune activator lipopolysaccharide (LPS) (Jones et al. 2017). We found that the role of inflammation differs after exposure to cigarette smoke condensate from that of the manifested disease in humans and propose that an initial suppressive immune response could contribute to the pathogenesis of COPD as a process starting earlier than we have assumed so far.

## Methods

### Ethics statement and donors

Human lung lobes were obtained from patients who underwent surgery for different reasons. The experiments were approved by the ethics committee of the Medical School Hannover (MHH, Hannover, Germany) and are in accordance with *The Code of Ethics of the World Medical Association*. All patients or their next of kin, caretakers, or guardians gave written informed consent for using lung tissue for research. Tissues from female and male donors were used. Diagnoses were tumor, fibrosis, emphysema, or pulmonary hypertension. Only tumor-free tissue as qualified by medical pathologists was used for experiments. One of the patients was older than 10 years; the others had an average age of 69 ± 15 years for the nine donors used for protein expression and 71 ± 14 years for the four donors used for gene expression (Table 1).

**Table 1:** List of human lung donor material used in this study. Characteristics of donors who provide lung material for this study and from whom protein expression data (A) or gene expression data (B) were extracted

(A) Donors for protein expression		
Donor number	Sex	Disease background
1	Female	Emphysema
2	Male	Pulmonary fibrosis
3	Male	Pulmonary fibrosis
4	Male	Pulmonary hypertension
5	Male	Pulmonary hypertension
6	Female	IPF
7	Male	Carcinoma
8	Male	Carcinoma
9	Female	Carcinoma
(B) Donors for gene expression		
Donor number	Sex	Disease background
7	Male	Carcinoma
8	Female	Carcinoma
9	Male	Carcinoma
10	Male	Carcinoma

### Preparation of human precision cut lung slices

Human precision cut lung slices (PCLS) were prepared as described before (Neuhaus et al. 2018). Briefly, human lung lobes were cannulated with a silicone tube and selected segments were inflated with 37 °C warm 1.5% low-gelling agarose (Fisher Scientific, Schwerte, Germany) in Dulbecco’s modified Eagle’s medium nutrient Mixture F-12 Ham (pH 7.2–7.4) with L-glutamine and 15 mM HEPES (DMEM). The lobe was kept on ice until the agarose was polymerized and cut into 3-cm-thick slabs. Tissue cores with a diameter of 8 mm were cut with a rotating sharpened coring tool and sliced with a microtome (Krumdieck tissue slicer, Alabama Research & Development, Munford, AL, USA) into about 250–300-µm-thick sections. The preparation was performed in Earle’s balanced salt solution (10 × EBSS). PCLS were subsequently washed three times with DMEM supplemented with 100 U/mL penicillin/100 µg/mL streptomycin and cultivated under normal cell culture conditions (37 °C, 5% CO_2_, 100% air humidity).

### Cigarette smoke condensate and LPS exposure of PCLS

CSC was prepared from Marlboro red cigarettes with 10 mg tar using RM/1G (Borgwaldt, Germany) smoking machine. The smoke of ten cigarettes was passed through a Cambridge filter (Borgwaldt, Hamburg, Germany) which was extracted using 10 mL dimethyl sulfoxide (DMSO) to generate CSC with a concentration of 10 mg/mL. PCLS were cultivated in 24-well plates. PCLS were stimulated using 450 µg/mL, 300 µg/mL, 150 µg/mL, 75 µg/mL, 37.5 µg/mL, and 9.4 µg/mL CSC diluted in DMEM. Similar CSC concentrations have been shown to be biologically relevant (Fields et al. 2005). Final concentration of CSC used for protein and gene expression analysis was selected as a non-toxic concentration of 9.4 µg/mL CSC equivalent to approx. 0.001 cigarettes on two PCLS. The solvent DMSO was used at its highest concentration of 3% as control for concentration assesment and approx. 0.09% as control for protein and gene expression analysis. PCLS were stimulated with 200 ng/mL LPS diluted in DMEM, as already published in Switalla et al. (2010). For LPS treatment, DMEM was used as control.

### Analysis of tissue viability

**Fig. 1:**
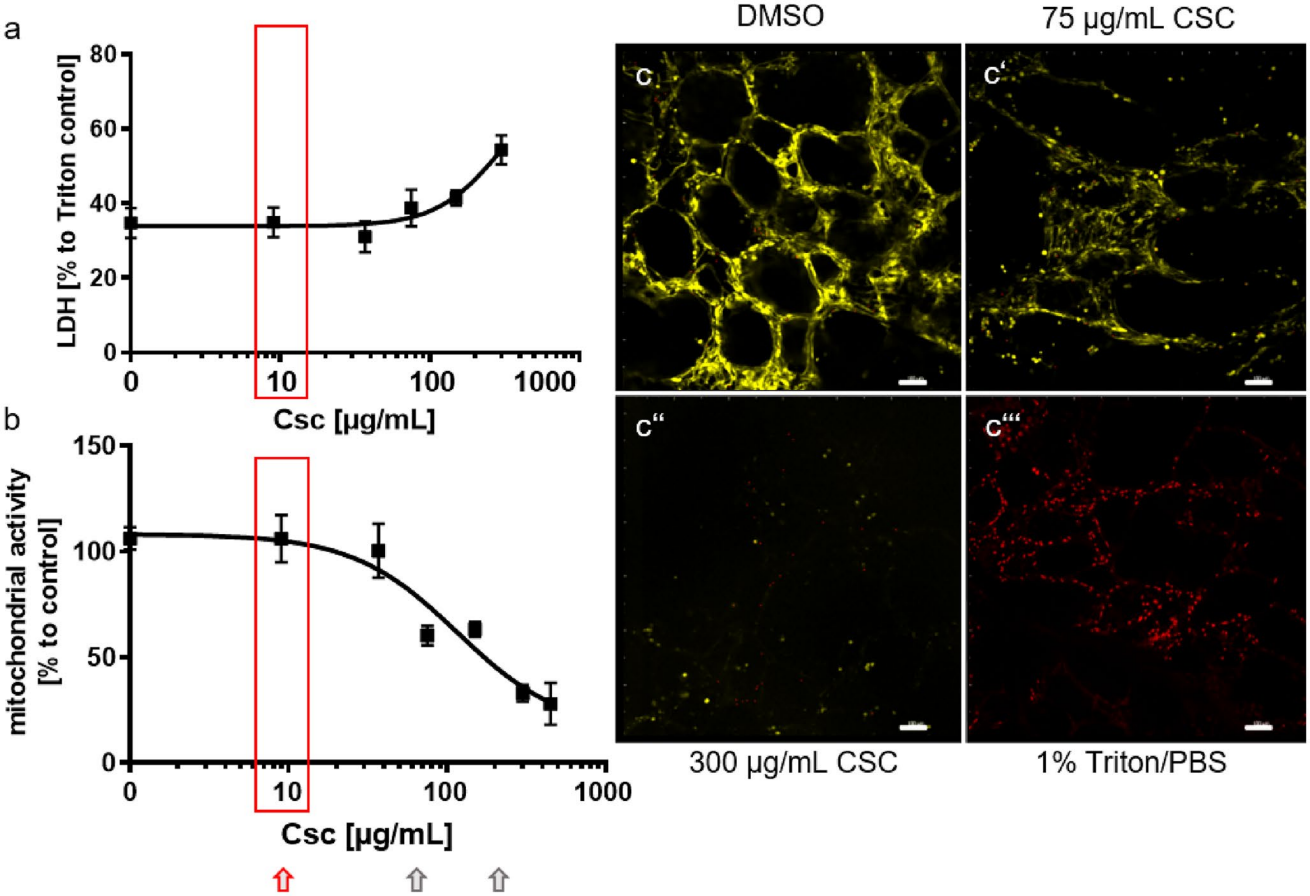
CSC induced cellular and mitochondrial toxicity in human PCLS. Human PCLS were exposed 24 h to different CSC concentrations (300 µg/mL, 150 µg/mL, 75 µg/mL, 37.5 µg/mL, and 9.4 µg/mL). (**a**) The cytotoxicity of CSC was measured by LDH assay. Increasing concentrations of CSC induced release of LDH from human tissue into supernatant as measured by LDH assay. (**b**) CSC also induced a decrease of metabolic activity as measured by WST-1 assay. (c–c’’’) LIVE/DEAD^®^ staining using Calcein-AM/EthD-1 and subsequent imaging by confocal microscopy also showed a loss of viable cells (yellow color, calcein staining) and an increase of the number of dead cells (red color, EthD-1 staining) in human PCLS after exposure to 75 µg/mL (c’) and 300 µg/mL CSC (c’’). DMSO at the highest concentration of 3% was used for all experiments as control (**c**). 1% Triton X-100 was used as positive control for maximum cytotoxicity (c’’’). Scale bar 100 µm. Toxic concentrations of CSC depicted in (c–c’’’) were marked with grey arrows in (**b**). Red boxes and red arrow marked the selected non-toxic CSC concentration of 9.4 µg/mL as used for gene and protein expression analysis. *N* = 7 for LDH and mitochondrial activity (**a**, **b**). *N* = 3 for LIVE/DEAD viability staining (c–c’’’)

Tissue viability was determined using LDH Cytotoxicity Assay Kit (Roche, Mannheim, Germany), LIVE/DEAD^®^ Viability/Cytotoxicity Kit (Thermo Fisher Scientific, Rockford, IL, USA), and cell proliferation assay WST-1 as described previously (Neuhaus et al. 2018). Briefly, LDH release assay was performed according to the manufacturer’s instructions. Shortly, LDH was detected in supernatants of tissue cultures using 50 µL of supernatant incubated with 50 µL of reagent for 20 min at RT in the dark. The maximum release of LDH was induced by 1 h treatment with 1% Triton-X 100. Absorption was detected with 492 nm and a reference wavelength of 630 nm. The absorption of the Triton-X 100 control was defined as 100%. The absorptions of all other samples were used to calculate the amount of released LDH described in percent. The solvent DMSO was used at its highest concentration of 3% as control. LIVE/DEAD^®^ viability staining was performed according to the manufacturer’s instructions. The LIVE/DEAD^®^ viability staining is a two-color fluorescence assay. It is based on the simultaneous identification of live and dead cells with two probes. The cell viability is identified by the intracellular esterase activity (depicted in yellow in Fig. 1(c–c’’’)) and plasma membrane integrity using calcein-AM and ethidium homodimer (EthD-1) (depicted in red in Fig. 1(c–c’’’)) which intercalates into the DNA of damaged cells. In brief, PCLS were incubated with 4 µM calcein-AM and 4 µM EthD-1 for 45 min at RT with 150 rpm on an orbital shaker. PCLS were washed with Dulbecco’s phosphate buffered saline (DPBS, 0.1 M sodium phosphate and 0.15 M NaCl, without Ca^2+^ and Mg^2+^, pH 7.4) (Lonza, Verviers, Belgium) and imaged by confocal laser scanning microscopy (LSM510 Meta, Zeiss, Germany). Of each PCLS, three independent locations were recorded generating 30-µm-thick 3D stacks (10 × , excitation wavelength 488 nm and 543 nm, emission filters BP 505–550 nm and LP 560 nm). Images were analyzed using the IMARIS 7.6.0 software. The solvent DMSO was used at its highest concentration of 3% as negative control. Triton-X 100 was used as control for maximum cytotoxicity. WST-1 assay was performed on the basis of manufacturer’s instructions. After cultivation, supernatants of PCLS cultures were removed and 250 µL of 1:10 WST-1 reagent dilution was added. Assay was performed under cell culture conditions for 1 h. Absorption of 100 µL of supernatant was detected with 450 nm and a reference wavelength of 630 nm. The solvent DMSO was again used at its highest concentration of 3% as control. LDH release and WST-1 was analyzed for seven donors. LIVE/DEAD staining was performed for three donors.

### Proteins and RNA

For protein analysis, the same donors were used as for gene expression analysis. Nevertheless, additional donors were performed for protein analysis as the expressions of some proteins were below the lower limit of detection of the ELISA. For measurement of cytokines, supernatants were collected from tissue cultures, supplemented with 0.2% protease inhibitor cocktail (P1860). Lung tissue was lysed using 1% Triton X-100 in DPBS supplemented with 0.2% protease inhibitor cocktail for 1 h at 4 °C. Supernatants and lysates were stored at − 80 °C until analysis. Procedures are described in detail before (Neuhaus et al. 2018). Enzyme-linked immunosorbent assay (ELISA) was performed according to the manufacturer’s instructions using R&D Systems DuoSet ELISA Kits (R&D Systems, Inc., Abingdon, UK). Protein content identified by ELISA of each sample was related to the total protein content (pg/mg total protein). The total protein content of each sample was analyzed with the Pierce^™^ BCA Protein Assay Kit (Thermo Fisher Scientific, Rockford, IL, USA) according to manufacturer’s instructions. Bovine serum albumin (included in the assay kit) was used for the standard curve. RNA was isolated from two tissue sections per sample as described by Niehof et al. (2017). For gene expression analysis, tissue of four donors was used as biological replicates. Overlap of the used donors from protein and gene expression analysis was described in Table 1. Briefly, immediately after cultivation, PCLS were transferred into tubes, snap frozen in liquid nitrogen, and stored at − 80 °C. For RNA isolation, tissue was homogenized in RLT lysis buffer (Qiagen, Hilden, Germany) using an Ultra-Turrax^®^ (T8, IKA, Staufen, Germany). The homogenate was transferred to phenol/chloroform, shaken, and centrifuged. Chloroform/isoamylalcohol was added, and suspension was shaken and centrifuged again. Isopropanol was added to the aqueous phase, and DNase treatment was performed. Finally, RNA was cleaned up using MagMax^™^ magnetic beads (Thermo Fisher Scientific, Dreieich, Germany). RNA concentration (A260) and purity (A260/A280 ratio) were measured by spectrophotometry (NanoDrop^™^ 2000 Spectrophotometer, software version 1.6.198, Thermo Fisher Scientific, Dreieich, Germany). RNA integrity number (RIN) was evaluated using an Agilent 2100 Bioanalyzer^®^ (Agilent Technologies, Ratingen, Germany). All RNA samples showed good quality as indicated by an A260/280 ratio around 2.00 and high RIN values between 9.0 and 9.9.

### Gene expression analysis and data evaluation

Expression was analyzed for the following 20 literature-based selected COPD-relevant genes: *ACTA2*, *COL1A1*, *CXCL11*, *CYP1A1*, *CYP1B1*, *FN1*, *HIF1A*, *IL1B*, *IL6*, *IL8*, *MMP12*, *MMP9*, *NFKB1*, *NOS2*, *SERPINA1*, *STAT1*, *STAT6*, *TIMP1*, *TNF*, and *VEGFA*. Gene expression analysis of cigarette smoke condensate–exposed samples and corresponding controls was performed using customized RT^2^ Profiler PCR Arrays (Qiagen, Hilden, Germany). *ACTB*, *B2M*, *HPRT1*, *LDHA*, and *RPLP0* were analyzed as potential reference genes. In the first step, cDNA synthesis was performed using the RT^2^ first strand kit (Qiagen), following the manufacturer’s instructions. Subsequently, RT^2^ profiler PCR arrays were conducted according to manufacturer’s instructions using the PCR system Applied Biosystems^®^ ViiA^™^7 (Thermo Fisher Scientific, Dreieich, Germany). An RNA equivalent of 4 ng was used per RTqPCR assay. Data analysis of exported “cycle threshold” (C_T_) values was performed based on the comparative ∆∆C_T_ method described by Schmittgen and Livak (2008). Reference genes for normalization were selected using NormFinder (Andersen et al. 2004) and geNorm algorithm (Vandesompele et al. 2002) as part of the GenEx Professional 6 Software (bioMCC, Freising, Germany). ΔC_T_ values, if normal distributed, were further checked for outliers according to Grubbs (Grubbs 1969). The fold change values were calculated for each gene describing the factor of up- or downregulation for each treatment group vs. corresponding control. Fold change (FC) < 0 were presented as (− 1)/FC. Genes that display FC values ≤  − 1.5 or ≥ 1.5 were considered as relevant, and statistical significance for FC values was checked with Student’s *t*-test with a probability of ≤ 0.05. Protein data in figures are given as mean ± *SD*, and gene expression data in figures are given as mean ± *SD*. Statistical analyses were performed by *t*-test using GraphPad Prism (Version 4.03, GraphPad, San Diego, CA, USA). Donors were only excluded if the positive control LPS did not induce a significant increase of pro-inflammatory cytokines such as IL-1β.

## Results

### Influence of CSC on viability of lung tissue was analyzed, and non-toxic concentrations were selected for gene and protein expression experiments

We investigated the impact of cigarette smoke condensate (CSC) on viability of human tissue from the lower respiratory tract ex vivo. Human lung tissue ex vivo was exposed to CSC for 24 h. Mitochondrial activity, release of lactate dehydrogenase, and intracellular esterase activity from human lung tissue slices were assessed by WST-1 assay, LDH activity assay, and LIVE/DEAD viability staining and imaging by confocal microscopy, respectively. CSC induced concentration-dependent loss of viability of human lung tissue ex vivo (Fig. 1). Lactate dehydrogenase release was slightly increased after 24 h at CSC concentrations above 75 µg/mL and strongly increased using 150 µg/mL CSC (Fig. 1(a)). Viability, measured by WST-1 assay, was slightly reduced above a concentration of 37.5 µg/mL CSC and decreased significantly at 75 µg/mL CSC, indicating a more sensitive viability assay for treatment of human PCLS with CSC (Fig. 1(b)). Esterase activity analyzed by LIVE/DEAD viability staining and imaging showed reduced viable tissue (yellow color in Fig. 1(c)) after exposure to 75 µg/mL CSC and nearly a complete loss of viable cells and an increase in dead cells in lung tissue ex vivo using 300 µg/mL CSC (Fig. 1(c’ and c’’)). The two concentrations depicted in the images of LIVE/DEAD viability staining after CSC treatment in Fig. 1(c) were marked with gray arrows below the graph of mitochondrial activity (Fig. 1(b)). Half maximal effective concentration (EC_50_) for lung tissue ex vivo exposed to CSC was 196 µg/mL using WST-1 assay and 303 µg/mL using LDH detection (Fig. 1(a, b)). LIVE/DEAD staining can only be analyzed semi-quantitative, so that the estimation of the half maximal effective concentration is less accurate compared to the other two viability assays. For the exposure experiments to determine protein and gene expression changes, a non-toxic CSC concentration of 9.4 µg/mL was selected and depicted with a red arrow below the graph of Fig. 1(b). The rationale of selecting a non-toxic CSC concentration behind was to distinguish primary immunomodulatory effects induced by CSC per se from secondary immunomodulatory effects due to cytotoxicity. High concentrations of CS might lead to a loss of cellular functions and induce secondary immune effects such as a decrease of cytokine production (Hoshino et al. 2001; Luppi et al. 2005; Ouyang et al. 2000; Soliman and Twigg 1992).

### LPS, but not CSC, induced significant changes on inflammatory immune reactions, whereas metabolic and ECM-associated proteins are increased by CSC, but not LPS

**Fig. 2:**
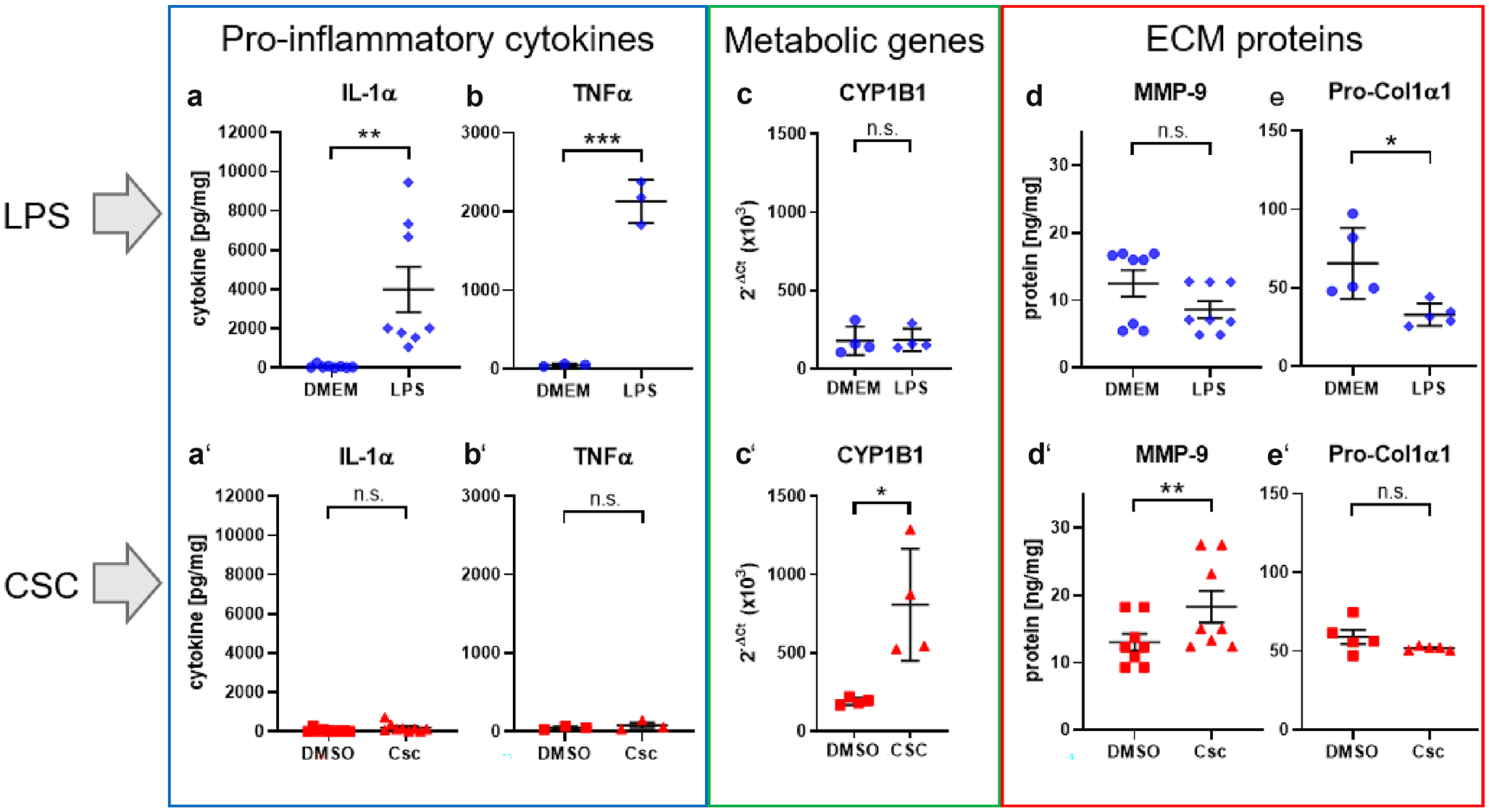
LPS, but not CSC, induced significant changes on inflammatory immune reactions, whereas metabolic and ECM-associated genes and proteins are increased by CSC, but not LPS. 200 ng/mL LPS induced release of pro-inflammatory cytokines IL-1α (**a**) and TNFα (**b**) from cells in viable human lung tissue slices. IL-1α (a’) and TNFα (b’) were unchanged after exposure to CSC. 9.4 µg/mL CSC altered release of protein MMP-9 (d’) associated to the extracellular matrix which was unchanged after stimulation with LPS (**d**). Additionally, 9.4 µg/mL CSC (c’), but not LPS (**c**), induced significant increase of metabolism-associated genes displayed as 2^−ΔCt^ values. Protein release was normalized to whole protein content and displayed in picogram of cytokine or protein per milligram of whole protein content. 2^−ΔCt^ values: Each donor was depicted as single data point ± *SD* tested by Student’s *t*-test, *n* = 4 donors, *p < 0.05 and **p < 0.005. Protein secretion: Each donor was depicted as single data point ± *SD* tested by Student’s *t*-test, *n* = 3–9 donors, *p < 0.05, **p < 0.005 and ***p < 0.001

Cigarette smoke exposure is known to be associated with increased degradation of extracellular matrix (ECM) proteins. We analyzed proteins of ECM in human viable lung tissue slices after exposure of lung tissue ex vivo to CSC in order to assess the influence of cigarette smoke tar phase components on ECM proteins. Matrix metalloproteinase-9 (MMP-9) was significantly induced (1.4-fold) by CSC (Fig. 2(d’)). Pro-collagen 1α1, extracellularly present, showed significant reduction after LPS exposure with 1.9-fold decrease (Fig. 2(e)). Pro-inflammatory cytokines are increased in bronchoalveolar lavage (BAL) and sputum samples of patients with COPD (Traves et al. 2002). Lipopolysaccharides (LPS) are used as mitogen to mimic increase of inflammatory cells into the lungs as observed in COPD (Lee et al. 2018). It is also well known to induce early innate immune responses in PCLS leading to production and release of pro-inflammatory cytokines and chemokines. In this study, pro-inflammatory cytokine protein levels IL-1α, TNFα, IL-8, and IL-1β were significantly increased by LPS treatment for 24 h with 66, 54, 1.7, and 16-fold, respectively, whereas a non-toxic concentration of CSC (9.4 µg/mL) showed no effect on pro-inflammatory cytokine protein levels IL-1α, TNFα, IL-8, and IL-1β in PCLS ex vivo (Figs. 2(a–b’) and 4). However, toxic concentrations of CSC induce tissue damage and thereby a loss of proteins from the cells. These proteins were degraded in the supernatant while cultivation time. This was confirmed by the detection of whole protein content (data not shown).

### Gene expression of literature-based COPD-relevant genes indicated cell damage by upregulation of genes involved in tissue injury and metabolic activity but less influence on pro-inflammatory cytokines in ex vivo human lung tissue after CSC exposure

**Fig. 3:**
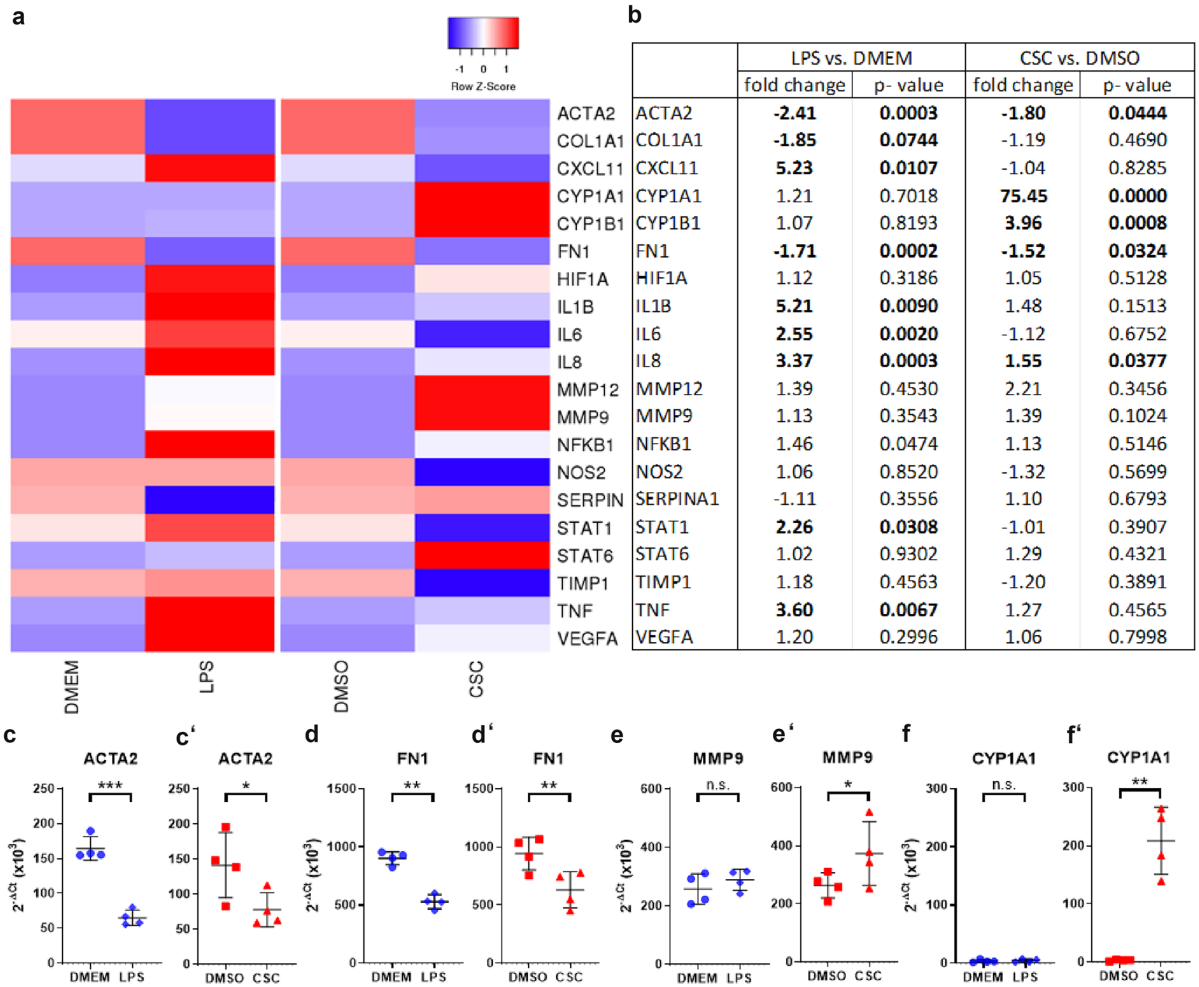
Gene expression of literature-based COPD-relevant genes indicated cell damage by upregulation of genes involved in tissue injury and metabolic activity. (**a**) Heat map of selected genes. Upregulated genes are represented in red, downregulated genes in blue and average in white. (**b**) Changes in gene expression in human PCLS after 200 ng/mL LPS or 9.4 µg/mL CSC exposure compared to corresponding control. Metabolic and ECM-associated differentially expressed genes indicate influence of CSC in expression profile. (c–f’) ECM-related markers in viable human lung tissue slices were changed by either CSC or LPS displayed as 2^−ΔCt^. FC ≥ 1.5 or ≤  − 1.5 and *p*-value < 0.05, RT^2^ Profiler PCR Assay from Qiagen. 2^−ΔCt^ values: Each donor was depicted as single data point ± *SD* tested by Student’s *t*-test, *n* = 4 donors, *p < 0.05 and **p < 0.005 and *** *p* < 0.001

**Fig. 4:**
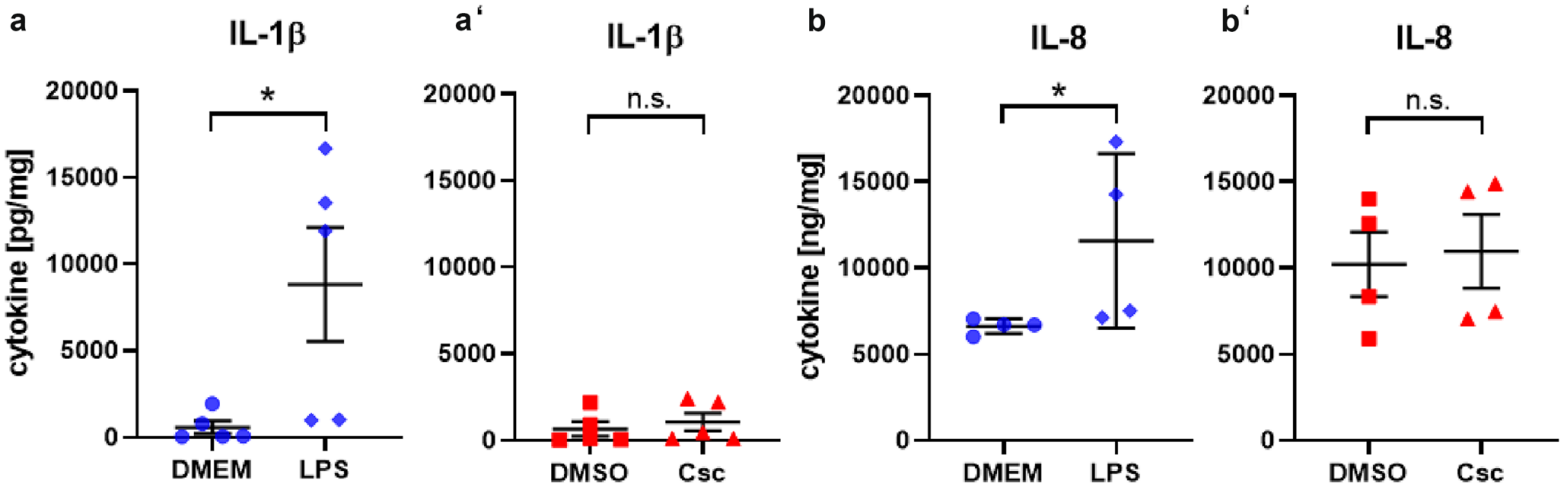
LPS, but not CSC, induced significant increase in inflammatory cytokines. Release of pro-inflammatory cytokines IL-1β (**a**) and IL-8 (**b**) from cells in viable human lung tissue slices was increased by 200 ng/mL LPS. 9.4 µg/mL CSC did not increase release of inflammatory cytokines IL-1β (a’) and IL-8 (b’) significantly. Cytokine release was normalized to whole protein content and displayed in picogram of cytokine per milligram of whole protein content. Each donor was depicted as single data point ± *SD* tested by Student’s *t*-test, *n* = 3–9 donors, *p < 0.05

**Fig. 5:**
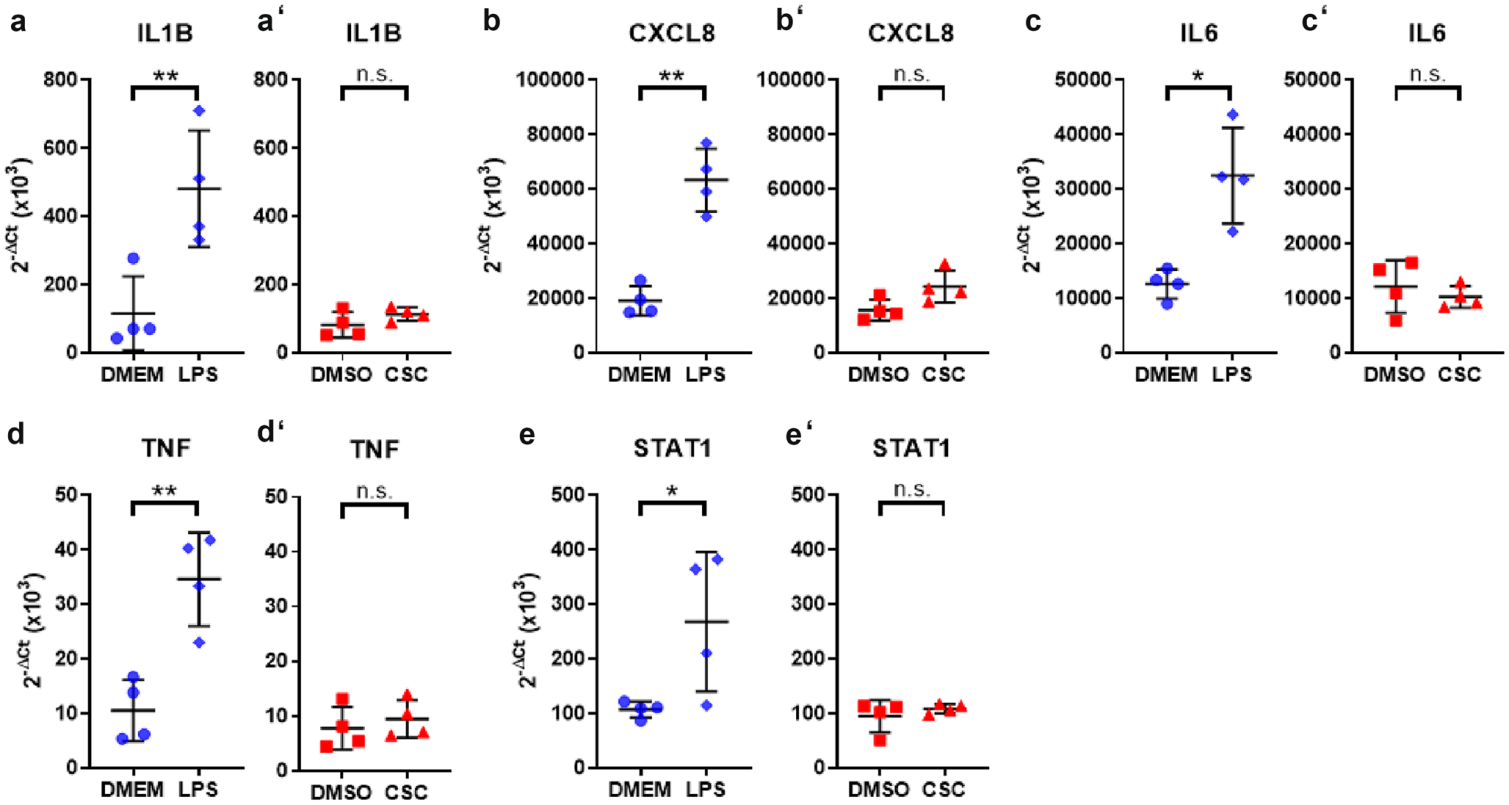
Examples of inflammatory differentially expressed genes by LPS exposure. (**a**–**e**) 200 ng/mL LPS, but not (a’–e’) 9.4 µg/mL CSC, induced significant increase of inflammatory genes displayed as 2^−ΔCt^ values. Each donor was depicted as single data point ± *SD* tested by Student’s *t*-test, *n* = 4 donors *p < 0.05 and **p < 0.005

**Fig. 6:**
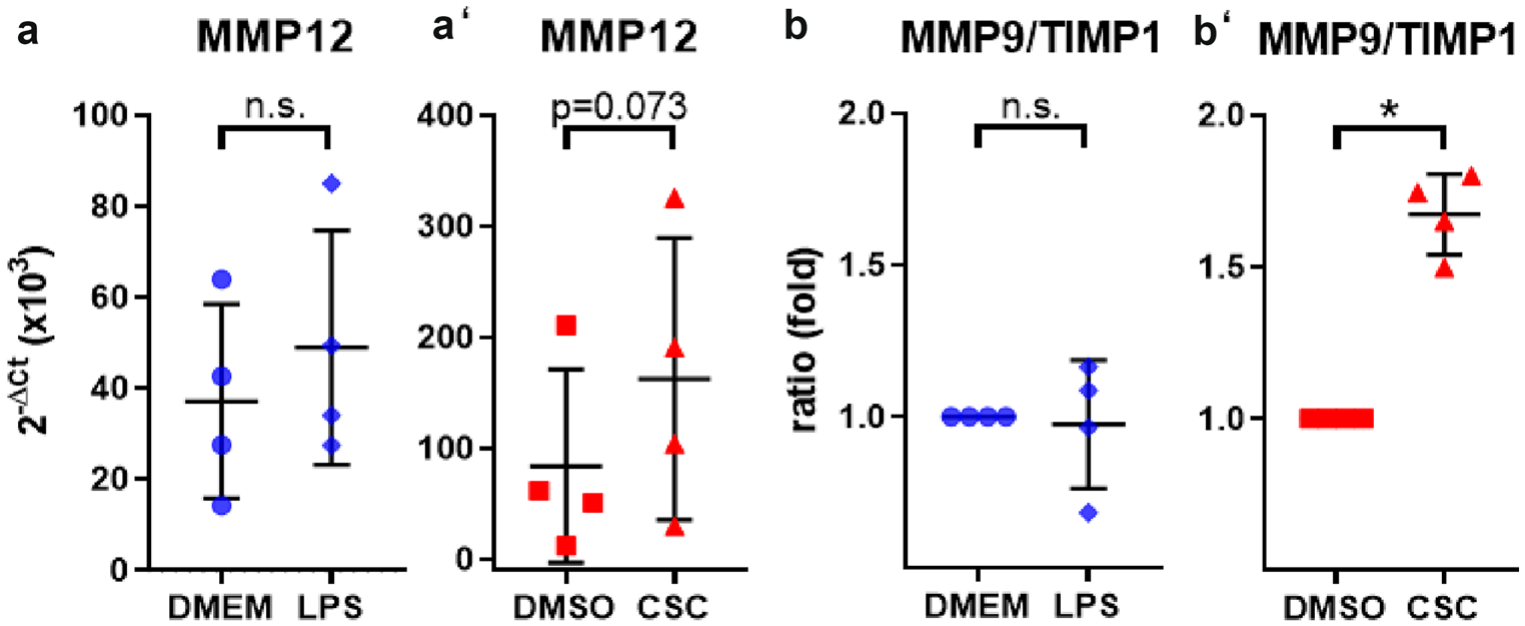
Examples of ECM-associated genes with changed expression levels by CSC exposure. Expression of ECM-related markers in cells in viable human lung tissue slices was changed by CSC. Shown in these gene expression results, 9.4 µg/mL CSC (a’, b’), but not 200 ng/mL LPS (**a**, **b**), induced partially significant increase of genes depicted as 2^−ΔCt^ values. Each donor was presented as single data point ± *SD* tested by Student’s *t*-test, *n* = 4 donors, *p < 0.05

We exposed human lung tissue ex vivo to CSC and LPS and analyzed changes in gene expression after 24 h by RT^2^ Profiler PCR Arrays. Heat map of gene expression analysis of selected genes demonstrated separation between gene expression profiles of LPS- vs DMEM- and CSC- vs DMSO-treated human viable PCLS (Fig. 3(a)). LPS increased fold change (FC) expression of genes involved in inflammatory processes such as *IL1B*, *IL6*, *TNF*, *IL8*, and *CXCL11*. Genes encoding transcription factors *NFKB1* and *STAT1* also show to be induced by LPS with *p*-values below 0.05, but *NFKB1* shows a fold change of 1.46 that is lower than the decided cutoff of 1.5. In contrast, gene transcripts associated with extracellular matrix were not regulated by LPS compared with medium control. CSC exposure induced significant induction of genes of the cytochrome P450 family, e.g. *CYP1B1*, and *CYP1A1*. ECM-relevant *MMP-12* involved in matrix formation showed an increased fold change but no statistical significance due to a high probability value. CSC exposure did not influence expression of genes of inflammatory pathways *IL1B*, *IL6*, *TNF* and *CXCL11*, except *IL8*, and significantly reduced expression of matrix building genes, e.g., *FN1* and *ACTA2* (Fig. 3(b)). In detail, Figs. 3(c–f’), 5, and 6 show 2^−ΔCt^ values for selected genes for individual donors. Markers of inflammation *TNF*, *IL1B*, *IL6*, *CXCL8*, and *STAT1* showed a significant increased gene expression in human PCLS after LPS exposure for 24 h compared with control with a 3.4-, 4.3-, 2.6-, 3.4-, and 2.5-fold increase, respectively (Figs. 4 and 5). CSC did not alter expression of these genes. In contrast, 2^−ΔCt^ values of *CYP1A1*, *CYP1B1*, *MMP9*, *ACTA2*, and *FN1* were changed after CSC exposure compared with control showing a 63-, 4.3-, and 1.4-fold increase as well as a 1.3- and 1.5-fold decrease, respectively (Figs. 3(c’–f’), 2(c, c’)). Results showed no influence of LPS on the expression of these genes except *ACTA2* and *FN1*, which is decreased (1.4- and 1.7-fold) by LPS exposure as well (Fig. 5(d)). In contrast, *MMP12* and *MMP9/TIMP1* ratio were highly expressed after CSC exposure compared with control showing a 1.9-fold increase for *MMP12* (Fig. 6(a’)). *MMP9/TIMP1* ratio, as a well-known marker in smokers and COPD patients, was not altered after LPS exposure compared with DMEM, but CSC treatment induced a 1.6-fold increased expression compared with DMSO (Fig. 6(b, b’)).

## Discussion

Data of initial effects of cigarette smoke on the distal healthy lung is limited (Ambrose and Barua 2004; Levitzky et al. 2008; Rom et al. 2013). In the presented study, distal lung tissue from human donors was exposed to CSC in comparison to LPS. We subsequently investigated pro-inflammatory markers and the transcriptome described as first pathological changes in the human lung of smokers LPS — but not CSC — induced inflammation associated genes and pro-inflammatory cytokines. CSC altered the extracellular matrix and metabolism.

Lung slices have the strong limitation of missing connection to the blood and lymphatics, but it is the only option to study human tissue. Inflammatory cytokines cause the pulmonary influx and activation of immune cells (Henjakovic et al. 2008; Switalla et al. 2010). An inflammation cascade of activation-influx-activation is absent in lung slices. Inflammatory responses in airways are similar to those observed in smokers and COPD patients (Baarsma et al. 2013; Kharitonov and Sjobring 2007; Vernooy et al. 2002). Exposure of human lung tissue to LPS ex vivo was earlier reported and led to an increase of pro-inflammatory cytokines and genes such as *IL1B, IL6, IL8,* and *TNF* (Neuhaus et al. 2017; Temann et al. 2017). In contrast to LPS, the inflammatory pattern on protein or gene levels was nearly unchanged after CSC exposure. At non-toxic CSC concentrations, the most susceptible cells might be the alveolar epithelial cells type II and macrophages as known for many noxae (Agarwal et al. 2014; Ritter et al. 2003; Zhao et al. 2017).

Cigarette smoke activates NFκB signalling in human lymphocytes (Ahn and Aggarwal 2005; Hasnis et al. 2007). Birrell et al. (2008) published that cigarette smoke inhibited the NFκB pathway in lung macrophages. The presented experiments found neither an upregulation of inflammatory genes such as NFκB nor an increased release of pro-inflammatory cytokines after exposure to CSC. This is in line with reported data (Birrell et al. 2008; Liu et al. 2008; Rastrick et al. 2013). Interestingly, ECM-associated genes and proteins were differently regulated by LPS and CSC. LPS reduced Pro-Col1α1 and MMP-9, whereas CSC significantly increased MMP-9. After CSC but not after LPS exposure increased expression of *MMP9*, *MMP12*, and *MMP9/TIMP1* was found. Altered ECM-associated genes and proteins demonstrated initial changes in the ECM induced by CSC in human lung tissue ex vivo. This was reported for smokers and COPD patients (Abdella et al. 2015; Boschetto et al. 2006; Demedts et al. 2006, Esa et al. 2016). Inflammation and elastolysis (O’Reilly et al. 2009) contribute to emphysema. Additionally, gene expressions of fibronectin *FN1* and α-smooth muscle actin *ACTA2* were reduced after CSC exposure in human PCLS. This altered expression of *FN1* and *ACTA2* has been published for smokers and COPD patients in airways and alveoli tissue (Karvonen et al. 2013; Wang et al. 2001).

Also, genes of the cytochrome p450 family were strongly differentially expressed by CSC (not LPS) exposure. The differentially expressed genes *CYP1A1* and *CYP1B1* are involved in drug metabolism and metabolic activation of pro-carcinogens (Spink et al. 1998). Polyaromatic hydrocarbons from cigarette smoke induce CYPs through the aryl hydrocarbon receptor (AhR) pathway (Kuehn et al. 2015; Lin et al. 2012). These observations fit to data from macrophages of smokers describing an increase of CYPs after years of smoking (Kamata et al. 2018; Woodruff et al. 2005). The tar phase of cigarette smoke is composed of toxic and carcinogenic substances damaging the lung of smokers (Talhout et al. 2011). Toxic destruction of lung parenchyma and release of intracellular proteins such as LDH was observed in the study (Fig. 1(a)) and elsewhere (Pickett et al. 2010; Ambrose and Barua 2004; Fields et al. 2005; Zhou et al. 2018). The missing pro-inflammatory response to CSC should not be caused by toxic effects of CSC since non-toxic concentrations of CSCs were applied. Other researchers observed that CSC induced anti-inflammatory responses, e.g., in dendritic cells (Cozen et al. 2004; Kroening et al. 2008; Vassallo et al. 2005). Nicotine as a component of CSC could have suppressed inflammatory reactions (Kroening et al. 2008; Razani-Boroujerdi et al. 2004; Tschernig et al. 2008).

Cigarette smoke can increase the susceptibility of smokers against viruses or induce exacerbations in COPD patients. Cigarette smoke exposure reduces immune reactions of host cells as shown by Cohen et al. and others (Arcavi and Benowitz 2004; Blake et al. 1988; Cohen et al. 1993; Feldman and Anderson 2013; Kark and Lebiush 1981; Kark et al. 1982). A main reason of exacerbations in COPD patients are viral infections, in most cases induced by rhinovirus, influenza, RSV, or parainfluenza (Hewitt et al. 2016). The combination of viral infections ex vivo with smoking could generate new insights.

In conclusion, our results show that CSC and LPS hit lung cells and lung tissue of COPD patients already early in smoking history.
